# Cytokine and Metabolomic Signatures of Mepolizumab Response Across Upper and Lower Airway Compartments in Severe Eosinophilic Asthma: An Exploratory Analysis

**DOI:** 10.3390/ph18111704

**Published:** 2025-11-10

**Authors:** Mauro Maniscalco, Pasquale Ambrosino, Claudio Candia, Antonino Di Stefano, Isabella Gnemmi, Martina Zappa, Nicolino Ambrosino, Dina Visca, Andrea Motta

**Affiliations:** 1 Istituti Clinici Scientifici Maugeri IRCCS, Pulmonary Rehabilitation Unit of Telese Terme Institute, 82037 Telese Terme, Italy; 2 Department of Clinical Medicine and Surgery, Federico II University, 80131 Naples, Italy; 3 Istituti Clinici Scientifici Maugeri IRCCS, Scientific Directorate of Telese Terme Institute, 82037 Telese Terme, Italy; pasquale.ambrosino@icsmaugeri.it; 4 Department of Biomedicine, Neurosciences and Advanced Diagnostics, University of Palermo, 90127 Palermo, Italy; claudio.candia@unipa.it; 5 Istituti Clinici Scientifici Maugeri IRCCS, Pulmonary Rehabilitation Unit of Veruno Institute, 28013 Gattico-Veruno, Italy; a.distefano89058@gmail.com (A.D.S.); isabella.gnemmi@icsmaugeri.it (I.G.); 6 Istituti Clinici Scientifici Maugeri IRCCS, Pulmonary Rehabilitation Unit of Tradate Institute, 21049 Tradate, Italy; martina.zappa@icsmaugeri.it (M.Z.); dina.visca@icsmaugeri.it (D.V.); 7 Istituti Clinici Scientifici Maugeri IRCCS, Pulmonary Rehabilitation Unit of Montescano Institute, 27040 Montescano, Italy; nicolino.ambrosino@icsmaugeri.it; 8 Department of Medicine and Surgery, Respiratory Diseases, University of Insubria, 21100 Varese, Italy; 9 Institute of Biomolecular Chemistry, National Research Council, 80078 Pozzuoli, Italy; andrea.motta@cnr.it

**Keywords:** asthma, chronic respiratory disease, chronic disease, oxidative stress, inflammation, mepolizumab, exercise, rehabilitation, disability, outcome

## Abstract

**Background**: Mepolizumab improves asthma control in severe eosinophilic asthma (SEA). However, its multidimensional effects on airway and systemic biomarkers are still incompletely understood. **Methods**: In this prospective study, 15 SEA patients were evaluated at baseline (T_0_), 6 (T_6_), and 12 months (T_12_) after starting mepolizumab. Lung function, FeNO values, asthma control, blood eosinophil count (BEC), cytokines, and metabolomic profiles (^1^H-NMR) were evaluated in serum, nasal secretions, and exhaled breath condensate (EBC). Univariate and multivariate (PCA, OPLS-DA) analyses were performed. **Results**: Mepolizumab reduced exacerbations, from a median of 2 at T_0_ to 0 at both T_6_ (*p* = 0.001) and T_12_ (*p* = 0.003). ACT improved from 18.7 ± 4.7 at baseline to 23.0 ± 2.8 at T_6_ (*p* = 0.026) and 23.4 ± 3.3 at T_12_ (*p* = 0.032), while FEV_1_ increased by 270 mL at T_6_ (*p* = 0.032) and remained stable at T_12_. Median BEC decreased from 450.0 (350.0–560.0) to 65.0 (50.0–87.5) cells/μL at T_6_ and to 50.0 (35.0–160.0) at T_12_ (*p* < 0.001), while FeNO showed a non-significant downward trend. IL-13 significantly decreased in serum and nasal secretions at T_6_ and T_12_, while IL-5 increased in nasal secretions at both timepoints and remained unchanged in serum. IL-2 showed opposite trends in serum and nasal samples, whereas GM-CSF and IFN-γ increased in nasal secretions at T_12_. Metabolomic profiling suggested compartment-specific changes, with decreased short-chain alcohols in EBC, increased amino acids in nasal secretions and serum at T_6_, and elevated pyruvate in serum at T_12_, although none reached statistical significance in univariate analysis. **Conclusions**: Mepolizumab induced consistent clinical, immunologic, and metabolic changes across compartments, supporting the use of integrated cytokine and ^1^H-NMR metabolomic profiling as a complementary approach for response assessment in SEA.

## 1. Introduction

Severe eosinophilic asthma (SEA) is a chronic respiratory condition characterized by persistent symptoms and poor disease control despite optimal adherence to maximal inhaled therapy [1]. SEA is associated with a higher risk of severe acute exacerbations and often requires maintenance oral corticosteroids (OCS) for disease control [2], frequently resulting in occupational disability [3,4] such as prolonged sick leave or reduced work capacity, and increased rehabilitation needs [5,6,7,8].

The pathogenesis of SEA involves type 2 (T2) immune polarization and eosinophil-driven airway inflammation, with eosinophil maturation, activation, and survival being critically regulated by interleukin-5 (IL-5) [9,10]. Moreover, eosinophilic asthma reflects T2 polarization of the adaptive response, while eosinophils act mainly in the effector phase. Basophils and group 2 innate lymphoid cells (ILC2s) can amplify this polarization through IL-4-dependent pathways, which sustain IgE production, mucus secretion, and airway hyperresponsiveness [11,12,13]. This immunologic framework provides the biological basis for precision-targeted therapies, as distinct biologics act on specific nodes of the T2 network, including IL-5 or its receptor and IL-4/IL-13 signaling [13,14]. Thus, the relatively recent development of targeted anti-IL-5 biological therapies has led to significant advances in the management of patients with SEA. Among these agents, mepolizumab, a humanized monoclonal antibody, has demonstrated the ability to improve asthma control, reduce the annual exacerbation rate, and decrease the reliance on maintenance OCS, thereby minimizing the associated adverse effects [15,16,17].

In parallel with the progress of biologic therapies, metabolomics has emerged as a valuable approach to better characterize respiratory diseases [18]. By identifying and quantifying small molecules generated during biological and inflammatory processes [19], metabolomics enables the mapping of metabolic pathways involved in disease pathophysiology, supporting both the diagnosis and assessment of treatment response [20]. Thus, nuclear magnetic resonance (NMR)-based metabolomics of biological fluids, including blood and exhaled breath condensate (EBC), has been proposed as a rapid and non-invasive strategy to support therapeutic decision-making in airway diseases [21,22,23], although standardization is still ongoing and NMR-based metabolomics is currently being explored in other respiratory and systemic conditions, such as chronic obstructive pulmonary disease, cystic fibrosis, and cardiovascular disorders [24,25,26].

Understanding how IL-5 blockade modulates both immune and metabolic pathways may clarify the multidimensional impact of biologic therapy in SEA. Therefore, this study aimed to assess the impact of mepolizumab on the nasal, bronchial, and systemic metabolomic profiles of consecutive patients with SEA and to explore the associations between these compartment-specific changes and clinical, inflammatory, and functional outcomes.

## 2. Results

Among 22 consecutive participants assessed for eligibility, seven were excluded for not meeting the inclusion criteria or for the presence of at least one exclusion criterion. Specifically, 2 were current smokers, 2 had a coexisting diagnosis of chronic obstructive pulmonary disease (COPD), and 1 had received systemic corticosteroid therapy within the 6 weeks preceding enrollment. Additionally, 1 patient reported a recent upper respiratory tract infection and 1 was undergoing treatment with another biologic agent. As a result, 15 patients (mean age 53.0 years; 26.7% males) were considered eligible and included in the final study cohort (Supplementary Figure S1). Of these, one participant was lost to follow-up. Baseline and follow-up characteristics are summarized in Table 1.

Five patients (33.3%) were former smokers, with a mean cumulative exposure of 18.8 pack-years. Thirteen individuals (86.7%) were classified as atopic based on clinical history and/or diagnostic testing. No participant reported a history of intolerance to ASA. All patients had been under treatment with an ICS/LABA at high dosage for at least six months before enrollment. No variation in the baseline inhalatory therapy was reported during the observational timeframe. Arterial hypertension was reported in 3 (20%) patients. No other relevant comorbidity was reported.

### 2.1. Impact of Mepolizumab on Functional, Inflammatory, and Patient-Reported Outcomes

Following the initiation of mepolizumab therapy, the baseline annual exacerbation rate significantly reduced to a median value of 0 both at T_6_ (*p* = 0.001) and T_12_ (*p* = 0.003), confirming a sustained anti-inflammatory effect on the airways (Table 2). At baseline, mean FEV_1_ was 2.02 ± 0.68 L, while FEV_1_% predicted was 71.9 ± 19.3%. Repeated-measures ANOVA with Bonferroni correction revealed a significant mean increase of 270 mL at T_6_, which persisted at T_12_ without further change (*p* > 0.999 for T_6_ vs. T_12_). Although FEV_1_% showed an increase over time, it did not reach statistical significance at T_6_ (*p* = 0.128 vs. T_0_), and only a trend toward significance was observed at T_12_ (*p* = 0.056 vs. T_0_).

Median FeNO values were 69.0 ppb (IQR: 25.0–111.0) at baseline, decreasing to 44.5 ppb at T_6_ and 31.0 ppb at T_12_, but without statistical significance across timepoints (*p* > 0.05 for all). In contrast, while BEC was markedly elevated at baseline, with a median of 450.0 cells/μL (IQR: 350.0–560.0), it showed a significant reduction at T_6_ (median: 65.0 cells/μL; IQR: 50.0–87.5; *p* < 0.001 vs. T_0_). This reduction persisted at T_12_, without any significant difference from T_6_ (*p* = 0.695 vs. T_6_).

ACT and ACQ-5 scores were also evaluated. At baseline, the mean ACT score was 18.7 ± 4.7, indicating poor asthma control. A significant improvement was observed at T_6_ (23.0 ± 2.8; *p* = 0.026), with values remaining stable at T_12_ (*p* > 0.999 for T_6_ vs. T_12_). Conversely, the mean ACQ-5 score at baseline was 1.23 ± 1.04, showing a numerical but non-significant improvement (*p* > 0.05 for all). Finally, a drop in the exacerbation rate was observed between baseline and follow-ups, reducing from a median of 2.0 (IQR: 2.0–3.0) to 0 (IQR: 0–1.0) at T_12_ (*p* = 0.003). Overall, the reduction in exacerbation frequency, improvement in asthma control, and sustained eosinophil decline together indicate a durable clinical and biological response to mepolizumab.

### 2.2. Impact of Mepolizumab on Cytokine Expression Across Biological Compartments

All comparisons between timepoints are detailed in Supplementary Table S2 and refer to cytokine concentrations measured across distinct biological compartments, namely the upper airways (nasal secretions), lower airways (EBC), and systemic circulation (serum). This design allowed a comprehensive assessment of compartment-specific immune changes over time.

**Figure 1:**
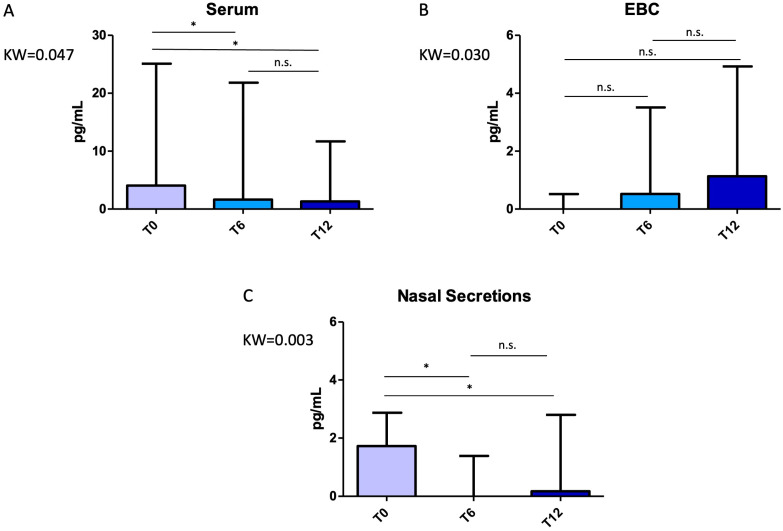
Variations in interleukin (IL)-13 concentrations across timepoints (T_0_, T_6_, T_12_) in different biological matrices: (**A**) serum, (**B**) exhaled breath condensate (EBC), (**C**) nasal secretions. Interestingly, a trend towards diminution was found in the serum and nasal matrices, while a numerical increase was found in the EBC, representing distal lung and airways. Albeit the genesis of this phenomenon is unclear, one could speculate that this might be a sort of compensatory mechanism, thus explaining the variable response in terms of FeNO that has been reported in the literature. Comparisons were performed with the Kruskal–Wallis test with Dunn’s post hoc correction. Error bars represent the maximum value. Significant *p*-values: Panel A, T_6_ vs. T_0_ *p* = 0.002, T_12_ vs. T_0_ *p* = 0.002; Panel C, T_6_ vs. T_0_ *p* = 0.007, T_12_ vs. T_0_ *p* = 0.015. Abbreviations: KW, Kruskal–Wallis *p*-value; n.s., not significant; *, significant at *p* < 0.05.

A reduction in IL-13 levels was observed in either serum or nasal secretions. Particularly, the median IL-13 serum value at T_0_ was 4.05 (IQR: 2.13–9.92) pg/mL, which lowered to 1.64 (IQR: 0.94–5.64) pg/mL and afterwards to 1.31 (IQR: 0.86–4.77) pg/mL, *p* = 0.002 for both comparisons. Similarly, the median IL-13 value in NL were 1.72 (IQR: 0.82–2.75) pg/mL at T_0_, 0 (IQR: 0–0.57) pg/mL at T_6_ and 0.17 (IQR: 0–1.05) pg/mL (*p* = 0.007 at T_0_ vs. T_6_, and *p* = 0.015 at T_0_ vs. T_12_). By contrast, a modest upward trend was noted in EBC (Figure 1).

**Figure 2:**
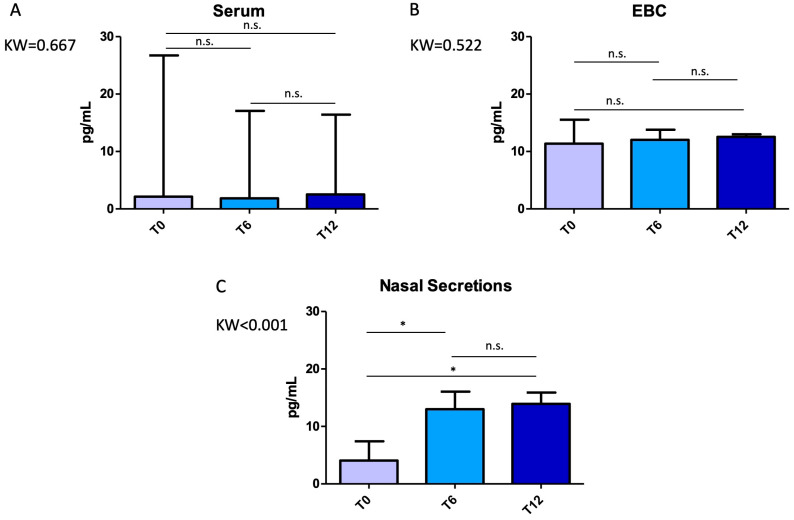
Variations in interleukin (IL)-5 concentrations across timepoints (T_0_, T_6_, T_12_) in different biological matrices: (**A**) serum, (**B**) exhaled breath condensate (EBC), (**C**) nasal secretions. It is noteworthy that IL-5 levels were found to significantly increase only at nasal level, although without reported increase in nasal symptoms among the study participants. Comparisons were performed with the Kruskal–Wallis test with Dunn’s post hoc correction. Error bars represent the maximum value. Significant *p*-values: Panel C, T_6_ vs. T_0_ *p* = 0.014, T_12_ vs. T_0_ *p* = 0.007. Abbreviations: KW, Kruskal–Wallis *p*-value; n.s., not significant; *, significant at *p* < 0.05.

Interestingly, IL-4 and eotaxin concentrations remained stable across all timepoints. In contrast, IL-5 levels significantly increased in concentrated nasal secretions at both T_6_ (median values: 12.99 (IQR: 11.68–14.58) pg/mL vs. 4.07 (IQR: 3.64–5.58) pg/mL at T_0_, *p* = 0.014) and T_12_ (median value: 13.92 (IQR: 13.12–15.11, *p* = 0.007) when compared to T_0_ (Figure 2). No significant change was detected in serum, EBC, or unconcentrated nasal samples (*p* > 0.05 for all).

Relevant changes were also detected for GM-CSF, whose concentration in nasal secretion increased significantly at T_12_ vs. T_0_. This was confirmed in both naïve (*p* = 0.039) and concentrated nasal samples (*p* = 0.014). A similar significant increase was observed for IFN-γ, although limited to naïve nasal secretions (*p* = 0.038 at T_12_ vs. T_0_ and *p* = 0.009 at T_12_ vs. T_6_). Finally, IL-2 exhibited a dual pattern, as its serum levels decreased significantly at T_12_ vs. T_0_ (*p* = 0.031), whereas a modest but statistically significant increase was observed in nasal secretion at T_12_ vs. T_6_ (*p* = 0.048, Supplementary Figure S2). These patterns collectively suggest distinct local immune dynamics between upper and systemic compartments.

### 2.3. Metabolomics

**Figure 3:**
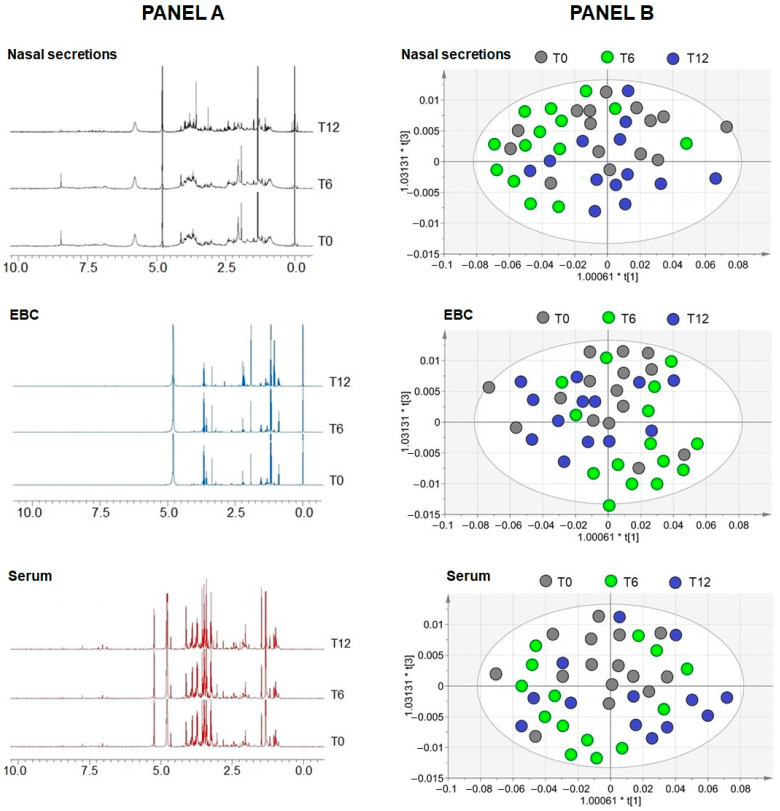
Representative one-dimensional (1D) proton nuclear magnetic resonance (NMR) spectra and scores plots of biological samples collected at baseline (T_0_, no treatment), 6 months (T_6_), and 12 months (T_12_) after the initiation of mepolizumab treatment. (**PANEL A**), three representative spectra are shown for each matrix for the three collection times. Chemical shifts of the peaks showing intensity variations are reported in the main text. (**PANEL B**), scores plots (each point represents a patient) showing the degree of separation of the models based on orthogonal projections to latent structures discriminant analysis (OPLS-DA) of metabolomic profiles obtained from biological samples at T_0_ (gray circles), T_6_ (green circles), and T_12_ (blue circles) after mepolizumab treatment. The models are described by quality parameters R^2^ = 0.43 and Q^2^ = 0.38 (nasal secretions, up panel B); R^2^ = 0.35 and Q^2^ = 0.30 (EBC, middle panel B); R^2^ = 0.37 and Q^2^ = 0.33 (serum, down panel B). They represent the goodness of fit of the model (R^2^), and the goodness of prediction (Q^2^). The labels t[1] and t[3] along the axes represent the scores (the first two partial least squares components) of the model, which are sufficient to build a satisfactory classification model. Altered metabolites in nasal secretion, EBC and serum samples and corresponding ^1^H chemical shifts, as detected by NMR spectroscopy are reported in Table 2. Since none of the discriminant metabolites reached statistical significance after analysis, their variation should be considered as a tendency to undergo modification throughout the T_0_, T_6_, and T_12_ treatment cycle.

One- and two-dimensional NMR experiments were used to characterize the metabolic profiles of nasal secretions, EBC, and serum collected at T_0_, T_6_, and T_12_ after initiation of mepolizumab therapy. Representative ^1^H spectra for each matrix are shown in Figure 3A, with metabolite identification based on 2D homonuclear and heteronuclear NMR analyses. For each matrix, MVA was performed. Initial PCA confirmed internal consistency and the absence of outliers, supporting data reliability. OPLS-DA was subsequently applied to evaluate treatment-related metabolic trajectories. Across the three biological compartments, OPLS-DA score plots (Figure 3B) showed varying degrees of class separation, particularly between T_0_ and post-treatment timepoints. In serum, T_0_ samples clustered separately from T_6_ and T_12_, with T_12_ appearing predominantly on the right side of the plot, suggesting a progressive treatment-related shift. In EBC, class separation was less pronounced, with partial inversion of T_6_ and T_12_ positions compared with serum. In nasal secretions, broader class dispersion was evident at T_0_, and partial overlap persisted, indicating higher biological heterogeneity. Despite this overlap, each matrix exhibited a distinct post-treatment metabolic pattern, with serum showing the clearest temporal trajectory and nasal secretions the greatest within-group variability.

Spectral regions affected by mucus (nasal) or salivary contamination (EBC) were excluded from analysis. In nasal secretions, T_0_ samples were characterized by higher threonine, valine, propionate, and methanol levels, T_6_ by elevated lysine, glutamate, aspartate, glutamine, and tyrosine while T_12_ by increased ethanol. In EBC, methanol, ethanol, and isopropanol predominated at T_0_, leucine and acetoin at T_6_, and alanine at T_12_. In serum, lactate and threonine dominated at T_0_, acetate, alanine and isoleucine increased at T_6_, and pyruvate distinguished T_12_. The ^1^H assignments of altered metabolites in the three biomatrices are reported in Table 2. Although these metabolites contributed to class separation in multivariate models, none reached statistical significance in univariate testing, underscoring the exploratory nature of these findings.

### 2.4. Correlations

No statistically significant correlations (*p* > 0.05) were detected between demographic, anthropometric, physiological, or clinical characteristics and cytokine or metabolomic data at any timepoint. Similarly, no significant correlations were found between altered metabolites across biological matrices (*p* ≈ 0.06). This likely reflects the limited sample size and consequent low statistical power (Table 1).

## 3. Discussion

In this prospective observational study, we investigated the clinical, immunological, and metabolomic effects of mepolizumab in SEA using a compartment-resolved approach. Overall, treatment was associated with sustained clinical improvement over 12 months, including fewer exacerbations, higher ACT scores, and a modest FEV_1_ gain. Blood eosinophils declined markedly, whereas FeNO showed only a limited downward trend. Cytokines changed in a compartment-dependent manner, with decreased IL-13 in serum and nasal secretions, a paradoxical rise in total IL-5 in concentrated nasal secretions, and divergent IL-2 dynamics, with delayed GM-CSF and IFN-γ increases in nasal secretions. In parallel, ^1^H-NMR metabolomics revealed compartment-specific metabolic shifts supported by multivariate separation, which we interpreted as exploratory. The novelty lies in the integrated, compartment-resolved immunometabolic profiling under anti-IL-5 therapy in a real-world cohort.

Our findings align with previous meta-analytical evidence [27] and further confirm in a real-world setting (where comorbidities and variable adherence may influence therapeutic outcomes) the long-term efficacy of anti-IL-5 therapy, thus highlighting the beneficial impact on asthma control and its modulatory effects on immunometabolic profiles. Specifically, the observed clinical improvements in our sample were paralleled by compartment-specific changes in selected cytokines and metabolites, suggesting a biologically consistent, though heterogeneous, immunological and metabolic response across different biological matrices. In this context, our pattern of changes is consistent with selective IL-5 pathway blockade, whereas IL-4/IL-13–directed agents would be expected to affect FeNO and IL-13 more directly, which helps interpret the compartment-specific signals observed.

From a clinical perspective, we documented a significant and sustained reduction in annual exacerbation rate following treatment initiation, with effects already evident at T_6_ and maintained after 12 months. These findings are consistent with phase II and III randomized controlled trials (RCTs), such as DREAM [28], MENSA [29], and MUSCA [30], which demonstrated significant reductions in exacerbation frequency and OCS dependence with mepolizumab treatment in similar patient populations. Accordingly, the improvement in lung function observed at 6 months, as indicated by the significant increase in FEV_1_, aligns with prior evidence from both RCTs [30] and real-world investigations, such as the REALITI-A study [31], indicating a modest but clinically relevant benefit in pulmonary function parameters among responders to anti-IL-5 therapy. Although the increase in FEV_1_% predicted did not reach statistical significance in our sample, the observed trend at T_12_, however, supports a favorable impact on airflow limitation over time. Moreover, consistent with the mechanism of action of mepolizumab [32], BEC also showed a marked and sustained reduction after treatment initiation in our study population. Interestingly, this biological response was also paralleled by improvements in ACT score already at T_6_, confirming the role of eosinophils as both a biomarker and a therapeutic target in T2-high asthma phenotypes [32]. In contrast, FeNO showed only a non-significant downward trend, in line with prior randomized trials and real-world studies [28,30,33]. This likely reflects its stronger association with IL-4/IL-13-driven inflammation rather than with IL-5-targeted interventions, confirming BEC as the most reliable biomarker of response [32]. It is nevertheless important to emphasize that changes in FeNO following biologic therapy have been shown to be largely independent of both clinical outcomes and the specific biologic agent employed [34].

An element of novelty in our study lies in the parallel assessment of cytokine concentrations across upper and lower airways as well as systemic circulation, which, partially aligning with previous evidence [35], revealed a complex and compartment-specific immunological response to biologics. In particular, IL-13 levels significantly decreased in both serum and nasal secretions, while remaining stable or increasing in EBC, consistent with the heterogeneous distribution of T2 inflammation along the respiratory tract [36,37]. Moreover, IL-4 levels did not show a significant change across timepoints in our cohort, which aligns with reports that mepolizumab does not modify circulating IL-4 concentrations over 24 weeks, while affecting other T2 mediators [38]. Unexpectedly, IL-5 levels increased in nasal secretions despite the reduction in BEC, a phenomenon previously described and attributed to the prolonged half-life of mepolizumab-IL-5 complexes and the inability of conventional immunoassays to discriminate between free and bound cytokine [39,40]. Interestingly, GM-CSF and IFN-γ exhibited delayed upregulation in nasal secretions after 12 months, which may reflect compensatory feedback mechanisms or even a shift toward innate/Th1-mediated responses after prolonged eosinophil suppression [41]. The divergent pattern of IL-2 expression, which decreased in serum but increased in nasal secretions, further highlights the need to consider local immune microenvironments when interpreting cytokine dynamics under biologic treatment.

Complementing the immunological findings, ^1^H-NMR-based metabolomic profiling revealed compartment-specific shifts in metabolic signatures following treatment. Although no single metabolite reached significance in univariate testing, multivariate models indicated a therapy-related shift. In EBC, short-chain alcohols decreased, whereas amino acid–related signals and pyruvate increased in serum and nasal secretions, consistent with downstream effects of eosinophil suppression [36,42]. OPLS-DA showed modest separation in airway matrices and a clearer temporal trajectory in serum, and we interpreted these patterns as exploratory. Conversely, the greater heterogeneity observed in nasal secretions and EBC may reflect the influence of local factors, including sampling variability, mucus or salivary interference, and compartment-specific kinetics of immunometabolic remodeling. This was most pronounced in nasal secretions, consistent with the widest intra-group dispersion reported in our results.

Within this framework, integrating noninvasive ^1^H-NMR metabolomics with clinical and cytokine readouts provides a more complete view of therapy-induced change, because it aligns metabolic and immunologic trajectories across compartments. The combined decline of IL-13 and the compartment-specific increase in total IL-5 in nasal secretions indicate localized immune adaptation and highlight the complex interplay between systemic and airway responses. These exploratory findings suggest mechanistic links between immune modulation and metabolic remodeling that warrant further investigation.

Taken together, our data provide a coherent and multidimensional picture of the effects of mepolizumab in SEA, encompassing clinical benefit, eosinophil depletion, cytokine modulation, and metabolic shifts. Although the sample size was limited and the analyses exploratory, this integrative approach offers translational insight into how anti-IL-5 therapy may reshape airway and systemic homeostasis.

### Limitations

Our protocol has several limitations that should be addressed. First, the small sample size limited statistical power and generalizability and increased the risk of type II error, particularly for metabolomics, which is sensitive to variability across biofluids [43]. As a result, metabolomic findings were framed as hypothesis-generating. However, although limited, the reported results highlight some relevant points in the treatment of severe eosinophilic asthma. Being aware of the small sample size (*n* = 15), we considered our report as an exploratory analysis. Second, although the study design included three distinct timepoints, the absence of a control group or active comparator limits causal inference for the observed immunometabolic changes. Consequently, temporal associations were interpreted with caution and were considered together with clinical and cytokine readouts. Information on occupational and specific environmental exposures was not collected in a standardized manner, which may limit interpretation of exposure-related effects. Moreover, multivariate analyses in small cohorts carry an inherent risk of overfitting, even with internal validation, and were therefore interpreted as exploratory. In this context, the lack of statistical significance in some of the univariate analyses of metabolomic and cytokine data may also reflect the complexity of sample handling and the intrinsic variability of biological fluids such as EBC and nasal secretions. Additionally, our assays did not distinguish free from total IL-5 across all matrices, which may influence interpretation of nasal measurements. Finally, although NMR spectroscopy offers high reproducibility and broad molecular coverage, the sensitivity is lower compared to mass spectrometry-based approaches, potentially limiting the detection of low-abundance metabolites [44]. Furthermore, the study was not powered to test formal mediation or prediction of clinical outcomes by immunometabolic markers, therefore observed associations with ACT and exacerbations were considered hypothesis-generating. In addition, this study was not designed to define actionable thresholds or clinical algorithms for metabolomics, and standardized pipelines with external validation are required before clinical use for individual monitoring.

## 4. Materials and Methods

### 4.1. Study Design and Population

We conducted a prospective, observational, multicenter cohort study of SEA adult individuals initiating mepolizumab, with 12-month follow-up. Consecutive patients referred to the Pulmonary Rehabilitation Units of the Istituti Clinici Scientifici Maugeri IRCCS in Telese Terme and Tradate, Italy, from September 2021 to September 2023, were screened for eligibility based on the following inclusion criteria: eligibility for mepolizumab treatment according to clinical practice; age between 18 and 75 years; diagnosis of severe eosinophilic refractory asthma, defined as peripheral blood eosinophil count (BEC) > 300 cells/μL and at least two documented exacerbations within the previous 12 months; and ongoing treatment with high daily doses of inhaled corticosteroids (ICS) combined with long-acting β_2_-agonists (LABA), plus at least one additional controller medication for a minimum of 12 months.

Exclusion criteria included: current smoking; diagnosis of other chronic pulmonary diseases; coexisting chronic rhinosinusitis with nasal polyps; use of systemic corticosteroids at any dose within the 6 weeks prior to enrollment; use of immunosuppressive therapies; receipt of live attenuated vaccines within 30 days prior to enrollment; current or recent history (within the last 5 years) of malignancy, except in cases of complete remission; diagnosis of eosinophilic granulomatosis with polyangiitis (EGPA); upper or lower respiratory tract infections within 30 days prior to signing informed consent or during the screening/run-in period; any clinically significant abnormalities identified during screening through physical examination, vital signs assessment, hematology, or clinical chemistry, which, in the opinion of the investigator, could pose a risk to patient safety or interfere with study outcomes or compliance. Additional exclusion criteria were: known immunodeficiency (primary or secondary); pregnancy; concurrent treatment with other biologics for asthma or other conditions (except for stable allergen immunotherapy, defined as an unchanged dose and regimen at screening); prior biologic therapy for asthma within 6 months before starting mepolizumab; planned surgical procedures during the study period; and participation in another interventional or post-authorization safety study.

### 4.2. Ethics, Registration, and Reporting Standards

The study was conducted in accordance with the STROBE (Strengthening the Reporting of Observational Studies in Epidemiology) guidelines, where applicable [45], and the trial was prospectively registered on ClinicalTrials.gov (Identifier: NCT05063981) in accordance with international standards for clinical research transparency and traceability. The study protocol was reviewed and approved by the competent Institutional Review Board of IRCCS Fondazione Pascale, Naples, Italy (approval number ICS9/20, on 18 November 2020) for the Institute of Telese Terme, and by the Institutional Ethics Committee of “Istituti Clinici Scientifici Maugeri” (approval number 2549/CE, on 28 April 2021) for the Institute of Tradate. Written informed consent was obtained from all participants prior to enrollment.

### 4.3. Study Protocol

The study lasted 24 months, including a 12-month enrollment period followed by a 12-month follow-up. Timepoints at 6 and 12 months were prespecified to capture early consolidation of treatment response and its one-year persistence, in alignment with routine follow up. Prior to inclusion, all participants underwent a clinical examination to assess eligibility. Clinical history was recorded, including age at asthma onset, smoking status, aspirin intolerance, number of exacerbations in the previous year, and asthma control level. Smoking status was recorded as never, former, or current, and current smokers were excluded. Occupational and specific environmental exposures were not systematically collected in this study. Evaluations were performed at baseline (T_0_), 6 (T_6_), and 12 months (T_12_) after initiation of treatment. At each timepoint, respiratory function was assessed and multiple biological matrices were collected to test markers of T2 inflammation and enable metabolomic profiling.

### 4.4. Measurements

Whenever applicable, all study procedures were performed for each participant at all timepoints (T_0_, T_6_, and T_12_), in dedicated rooms maintained at a constant temperature of 23 °C. The Asthma Control Test (ACT) and the 5-item Asthma Control Questionnaire (ACQ-5) were administered at each visit to assess the individual level of asthma control over time, using standardized and validated clinical instruments [46,47].

Exacerbations were defined as acute worsening of symptoms such as cough, sputum, dyspnea, requiring a treatment course with oral steroids, according to the latest GINA report [1]. The number of exacerbations was reported at baseline (referring to the 12 months prior to enrolment) and at each timepoint (referring to the previous six months of observation).

Moreover, in accordance with the American Thoracic Society/European Respiratory Society (ATS/ERS) guidelines [48,49] and using a fully automated equipment (Vmax^®^ Encore, Vyasis Healthcare, Milan, Italy), spirometry was performed in all participants to measure forced expiratory volume in one second (FEV_1_), reported both as absolute values and as percentage of predicted values.

Markers of T2 inflammation, including BEC and fractional exhaled nitric oxide (FeNO), were measured. FeNO was assessed by a trained operator and expressed in parts per billion (ppb), using an automated electrochemical analyzer (HypAir FeNO^®^, Médisoft, Sorinnes, Belgium), according to a standardized procedure previously described in detail [50,51].

Finally, biological matrices, including nasal secretions, EBC, and serum, were collected for cytokine quantification. Thus, the concentration of IL-2, IL-3, IL-4, IL-5, IL-13, transforming growth factor beta (TGF-β), eotaxin, granulocyte-macrophage colony-stimulating factor (GM-CSF), interferon gamma (IFN-γ), and thymic stromal Lymphopoietin (TSLP) was measured using commercially available enzyme-linked immunosorbent assay (ELISA) kits, as detailed in Supplementary Table S1. At each timepoint, metabolomic profiling of the collected biological samples was also carried out using NMR spectroscopy as detailed below.

### 4.5. Sample Collection

As previously reported [52], nasal blown secretions were collected, immediately processed, and centrifuged to obtain the supernatants, which were then stored at −80 °C for the subsequent analysis of soluble mediators.

EBC was collected using the Turbo-DECCS^TM^ condenser (Medivac, Pilastrello, Parma, Italy), maintained at a constant temperature of −5.0 ± 1.0 °C. Participants breathed at tidal volume through a mouthpiece for 15 min while comfortably seated and wearing a nose clip. An average volume of 2.0 ± 0.3 mL of EBC was obtained per subject. Samples were immediately transferred to polypropylene tubes, frozen in dry ice, and stored at −80 °C until NMR analysis. The absence of salivary contamination was confirmed by measuring alpha-amylase activity.

Peripheral blood was also collected for eosinophil percentage determination, while serum and plasma aliquots were stored at −20 °C for the quantification of circulating biomarkers.

### 4.6. NMR Sample Preparation and Acquisition

Biological samples were rapidly thawed and centrifuged. To enable field frequency locking, 70 μL of a deuterated water (^2^H_2_O) solution containing 0.1 mmol/L sodium 3-(trimethylsilyl)-[2,2,3,3-^2^H_4_]propionate (TSP), used as an internal chemical shift reference for ^1^H NMR spectra, and 3 mmol/L sodium azide, used as a bacteriostatic agent, were added to 630 μL of nasal secretions or EBC, reaching a final volume of 700 μL. Spectra were acquired at the Institute of Biomolecular Chemistry, National Research Council (CNR), Pozzuoli, Italy using a Bruker^®^ Avance III 600 MHz NMR spectrometer (Bruker BioSpin GmbH^®^, Karlsruhe, Germany) equipped with a CryoProb^TM^ and a temperature-controlled, automated 24-position sample changer managed through ICON-NMR^TM^ software (TOPSPIN^TM^, version 3.60, Bruker BioSpin GmbH^®^). All samples were maintained at a constant temperature of 300 K (27 °C). One-dimensional (1D) proton spectra were recorded using the excitation sculpting pulse sequence for a water suppression [20]. In addition, two-dimensional (2D) homonuclear (^1^H−^1^H clean TOCSY) and heteronuclear (^1^H–^13^C HSQC) experiments were performed to determine spin connectivity and proton-carbon correlations, respectively. For serum samples, 1D spectra were acquired with a T_2_ relaxation filter based on the Carr–Purcell–Meiboom–Gill (CPMG) sequence, designed to attenuate broad macromolecular signals and enhance low-molecular-weight metabolite visibility [20].

All spectra were referenced to the internal standard TSP (0.1 mmol/L), with the signal calibrated at δ = 0.00 ppm. HSQC spectra were referenced using the α-glucose doublet, set at 5.24 ppm (^1^H) and 93.10 ppm (^13^C).

### 4.7. Statistical Analysis

Clinical data were analyzed using the IBM SPSS Statistics software, version 29.0 (IBM Corp., Chicago, IL, USA). The Shapiro–Wilk test was applied to assess the normality of distribution of continuous variables. Normally distributed continuous variables were expressed as mean ± standard deviation (SD), whereas non-normally distributed data were reported as median and interquartile range (IQR). Multiple comparisons across timepoints were conducted using repeated-measures ANOVA with Bonferroni-adjusted post hoc testing or, where appropriate, Friedman test followed by pairwise post hoc comparisons. Categorical variables were summarized as relative frequencies and analyzed using Pearson chi-square test or Fisher exact test, as appropriate. Correlations between variables were assessed using Pearson or Spearman correlation coefficients, and subsequently examined through multivariate linear regression models. Given the limited sample, all inferential analyses were treated as exploratory and results were interpreted with caution, emphasizing direction and consistency across endpoints rather than isolated *p*-values.

### 4.8. Multivariate Data Analysis

Metabolomics was planned as a complementary, noninvasive readout to interpret immunologic and clinical changes across compartments over time. Multivariate data analysis (MVA) was performed to assess group discrimination among samples. Proton NMR (^1^H-NMR) spectra, acquired over the range of 9.0 to 0.60 ppm, were automatically divided into 420 equally spaced bins (Δδ = 0.02 ppm), referred to as “buckets”, and subsequently integrated using the AMIX 3.9.15 software package (Bruker BioSpin GmbH^®^, Rheinstetten, Germany). The spectral region between 5.0 and 4.6 ppm, corresponding to the residual water signal, was excluded. To reduce variability due to sample dilution, each bucket integral was normalized to the total spectral area. The resulting dataset was organized into an X matrix (predictor variables) and imported into SIMCA-P+ version 14 (Umetrics, Umeå, Sweden) for statistical modeling. Prior to analysis, data were scaled using the Pareto method to enhance moderate variance while reducing the influence of high-intensity signals. Principal Component Analysis (PCA) was initially applied as an unsupervised method to explore intrinsic data structure and to identify potential outliers. Once sample homogeneity was confirmed, Orthogonal Partial Least Squares Discriminant Analysis (OPLS-DA) was performed to assess class separation and extract discriminatory metabolite features. A Y matrix (response variable) was constructed by assigning dummy variables to each sample class. Regression models were computed between the X and Y matrices, and model performance was evaluated through R^2^ (goodness-of-fit) and Q^2^ (predictive ability), supported by 7-fold cross-validation, 800-fold permutation testing, and cross-validated analysis of variance (CV-ANOVA) to assess statistical robustness and rule out overfitting.

## 5. Conclusions

In this prospective real-world cohort of SEA patients, integrated clinical, cytokine, and ^1^H-NMR metabolomic profiling revealed compartment-resolved effects of mepolizumab. We observed sustained clinical improvement with a marked reduction in blood eosinophils and compartment-specific cytokine changes, together with exploratory metabolomic shifts consistent with selective IL-5 pathway blockade. These findings provide translational insight into how anti-IL-5 therapy shapes immunometabolic networks across airway and systemic compartments. Larger controlled studies are needed to validate these signals and to test whether integrated profiles can refine monitoring and treatment decisions.

## Figures and Tables

**Table 1:** Demographic, anthropometric, physiological and clinical characteristics of participants at T_0_ (baseline), T_6_ (6 months), and T_12_ (12 months). Data are expressed as mean ± standard deviation or median (interquartile range, IQR), unless otherwise specified.

Variables	T_0_ (*n* = 15)	T_6_ (*n* = 14)	T_12_ (*n =* 13)	T_0_ vs. T_6_	T_0_ vs. T_12_	T_6_ vs. T_12_
Age, years	53.0 ± 11.9	-	-	-	-	-
AAO, years	34.0 ± 19.8	-	-	-	-	-
Males, *n* (%)	4 (26.7)	-	-	-	-	-
BMI, kg/m^2^	26.5 ± 6.6	-	-	-	-	-
No. Exac.	2.0 (2.0–3.0)	0 (0–1.0)	0 (0–1.0)	**0.001**	**0.003**	>0.999
FEV_1_ (L)	2.02 ± 0.68	2.29 ± 0.90	2.25 ± 0.80	**0.032**	**0.043**	>0.999
FEV_1_ (%)	71.9 ± 19.3	78.4 ± 21.4	81.3 ± 18.2	0.128	0.056	>0.999
FeNO (ppb)	69.0 (25.0–111.0)	44.5 (25.8–94.8)	31.0 (15.0–60.0)	>0.999	0.096	0.199
ACT	18.7 ± 4.7	23.0 ± 2.8	23.4 ± 3.3	**0.026**	**0.032**	>0.999
ACQ-5	1.23 ± 1.04	0.54 ± 0.86	0.45 ± 0.65	0.192	0.059	>0.999
Eosinophils, cells/μL	450.0 (350.0–560.0)	65.0 (50.0–87.5)	50.0 (35.0–160.0)	**<0.001**	**<0.001**	0.695

Abbreviations: AAO, age at asthma onset; BMI, body mass index; Exac, exacerbations; FEV_1_, forced expiratory volume in 1 s; FeNO, fractional exhaled nitric oxide; ACT, asthma control test; ACQ-5, asthma control questionnaire-5; ppb, parts per billion; μL, microliter. Statistically significant *p* values are in bold.

**Table 2:** Identified signals from altered metabolites in nasal secretion, EBC and serum samples and corresponding ^1^H chemical shifts, as detected by NMR spectroscopy.

Metabolites	Moieties	δ ^1^H (ppm) and Multiplicity
Aspartate	αCH	3.85 (dd)
	βCH	2.67 (dd)
	β’CH3	2.82 (dd)
Acetate	βCH_3_	1.90 (s)
Acetoin	CH	4.42 (q)
	CH_3_	2.21 (s)
	CH_3_	1.38 (d)
Alanine	βCH_3_	1.46 (d)
	CH	3.77 (m)
Ethanol	CH_3_	1.17 (t)
	CH_2_	3.64 (q)
Glutamate	αCH	3.79 (t)
	β,β’CH	2.03 (dt)
	γCH_2_	2.30 (t)
Glutamine	αCH	3.77 (t)
	βCH_2_	2.10 (c)
	γCH_2_	2.40 (c)
Isoleucine	αCH	3.68 (m)
	βCH	1.95 (m)
	γ’CH_3_	1.00 (m)
	δCH_3_	0.92 (t)
Isopropanol	CH	4.02 (m)
	(CH_3_)_2_	1.18 (d)
Lactate	βCH_3_	1.34 (d)
	αCH	4.11 (q)
Leucine	αCH	3.60 (t)
	βCH_2_	1.70 (m)
	δ,δ’CH_3_	0.95 (d)
Propionate	αCH_2_	2.19 (q)
	βCH_3_	1.06 (t)
Pyruvate	CH_3_	2.36 (s)
Threonine	αCH	3.62 (d)
	βCH	4.22 (m)
	γCH_3_	1.33 (d)
Tyrosine	C_3,5_H, Ring	6.88 (d)
	C_2,6_H, Ring	7.17 (d)
Valine	αCH	3.60 (d)
	βCH	2.26 (m)
	γCH_3_	0.98 (d)
	γ’CH_3_	1.03 (d)

Abbreviations: c, complex; d, doublet; dd, double doublet; dt, double triplet; m, multiplet; q, quartet; s, singlet; t, triplet.

## Data Availability

The data supporting the findings of this study are available from the corresponding author upon reasonable request due to privacy/ethical restrictions.

## Supplementary Materials

The following supporting information can be downloaded at: https://www.mdpi.com/article/10.3390/ph18111704/s1, Supplementary Table S1, Enzyme-Linked Immunosorbent Assays (ELISA) tests used for the quantitative determination of biomarkers included in the study; Supplementary Table S2, Comparisons of baseline and follow-up cytokine expression across biological compartments in patients with severe eosinophilic asthma at T_0_ (baseline), T_6_ (6 months), and T_12_ (12 months); Supplementary Figure S1, Schematic flowchart of the enrollment process; Supplementary Figure S2, Variations in Interleukin (IL)-2, IL-3, IL-4, Granulocyte-Macrophage Colony-Stimulating Factor (GM-CSF), Thymic Stromal Lymphopoietin (TSLP), and Transforming Growth Factor-beta (TGF-β) across timepoints (T_0_, T_6_, T_12_) in different biological matrices, including serum, exhaled breath condensate (EBC), nasal secretions, and concentrated nasal secretions. KW: Kruskal–Wallis *p*-values.
