# Supplementary material for: Cytokine and Metabolomic Signatures of Mepolizumab Response Across Upper and Lower Airway Compartments in Severe Eosinophilic Asthma: An Exploratory Analysis
Source: Pharmaceuticals (Basel). 2025 Nov 10;18(11):1704. doi: 10.3390/ph18111704 (PMC12655059; doi:10.3390/ph18111704)
Supplement: Supplementary file 1 [file pharmaceuticals-18-01704-s001.zip › pharmaceuticals-3903679-SI.pdf]

Supplementary Material

Cytokine and Metabolomic Signatures of Mepolizumab Response Across Upper and Lower Airway Compartments in Severe Eosinophilic Asthma: An Exploratory Analysis

Mauro Maniscalco [1,2,*], Pasquale Ambrosino [3], Claudio Candia [4], Antonino Di Stefano [5], Isabella Gnemmi [5], Martina Zappa [6], Nicolino Ambrosino [7], Dina Visca [6,8] and Andrea Motta [9]

1 Istituti Clinici Scientifici Maugeri IRCCS, Pulmonary Rehabilitation Unit of Telese Terme Institute, 82037 Telese Terme, Italy
2 Department of Clinical Medicine and Surgery, Federico II University, 80131 Naples, Italy
3 Istituti Clinici Scientifici Maugeri IRCCS, Scientific Directorate of Telese Terme Institute, 82037 Telese Terme, Italy; pasquale.ambrosino@icsmaugeri.it
4 Department of Biomedicine, Neurosciences and Advanced Diagnostics, University of Palermo, 90127 Palermo, Italy; claudio.candia@unipa.it
5 Istituti Clinici Scientifici Maugeri IRCCS, Pulmonary Rehabilitation Unit of Veruno Institute, 28013 Gattico-Veruno, Italy; a.distefano89058@gmail.com (A.D.S.); isabella.gnemmi@icsmaugeri.it (I.G.)
6 Istituti Clinici Scientifici Maugeri IRCCS, Pulmonary Rehabilitation Unit of Tradate Institute, 21049 Tradate, Italy; martina.zappa@icsmaugeri.it (M.Z.); dina.visca@icsmaugeri.it (D.V.)
7 Istituti Clinici Scientifici Maugeri IRCCS, Pulmonary Rehabilitation Unit of Montescano Institute, 27040 Montescano, Italy; nicolino.ambrosino@icsmaugeri.it
8 Department of Medicine and Surgery, Respiratory Diseases, University of Insubria, 21100 Varese, Italy
9 Institute of Biomolecular Chemistry, National Research Council, 80078 Pozzuoli, Italy; andrea.motta@cnr.it
* Correspondence: mauro.maniscalco@icsmaugeri.it

Supplemental Table S1. Enzyme-Linked Immunosorbent Assays (ELISA) tests used for the quantitative determination of biomarkers included in the study.

Molecules	Manufacturer (code)	Lower detection limit	Type of samples
IL-2	Invitrogen™ (BMS221INST)	< 2.3 pg/mL	Serum/NS/EBC
IL-3	Invitrogen™ (KHC0031)	< 1.0 pg/mL	Serum/NS/EBC
IL-4	Invitrogen™ (BMS225-2)	< 1.3 pg/mL	Serum/NS/EBC
IL-5	Invitrogen™ (BMS278INST)	< 1.45 pg/mL	Serum/NS/EBC
IL-13	Invitrogen™ BMS231INST)	< 0.99 pg/mL	Serum/NS/EBC
IL-33	Invitrogen™ (BMS2048)	< 0.9 pg/mL	Serum/NS/EBC
TGF-β	Invitrogen™ BMS249-4)	< 8.6 pg/mL	Serum/NS/EBC
Eotaxin	Invitrogen™ (KAC2231)	< 2.2 pg/mL	Serum/NS/EBC
GM-CSF	Invitrogen™ (KHC2011)	< 3.0 pg/mL	Serum/NS/EBC
IFN-γ	Invitrogen™ (BMS228)	< 0.99 pg/mL	Serum/NS/EBC
TSLP	Invitrogen™ (EHTSLP)	< 3.0 pg/mL	Serum/NS/EBC

Abbreviations: IL: Interleukin; TGF-β: Transforming Growth Factor beta; EBC: Exhaled Breath Condensate; NS: Nasal Secretion; GM-CSF: Granulocyte-Macrophage Colony-Stimulating Factor; IFN-γ: Interferon gamma; TSLP: Thymic Stromal Lymphopoietin; pg: picograms; mL: millilitres. For IL-5, values reflect total IL-5 measured by the assay, which detects antibody-bound and free cytokine. In addition, the assay does not separate free from total IL-5 in all matrices.

Supplemental Table S2. Comparisons of baseline and follow-up cytokine expression across biological compartments in patients with severe eosinophilic asthma at T_0 (baseline), T_6 (6 months), and T_{12} (12 months).

	**P value (T_0 *vs.* T_6)**	**P value (T_0 *vs.* T_{12})**	**P value (T_6 *vs.* T_{12})**
		**Eotaxin**	
Serum	0.123	0.105	0.425
Nasal secretions	0.640	0.640	0.570
EBC	0.437	0.437	0.750
		**IL-4**	
Serum	0.143	0.678	0.812
Nasal secretions	1.0	0.109	0.468
EBC	0.125	0.812	0.250
		**IL-5**	
Serum	0.247	0.845	0.742
Nasal secretions	**0.014**	**0.007**	0.195
EBC	1.0	0.125	1.0
		**IL-13**	
Serum	**0.002**	**0.002**	0.400
Nasal secretions	**0.007**	**0.015**	0.207
EBC	0.250	0.062	0.750
		**GM-CSF**	
Serum	1.0	0.845	0.483
Nasal secretions	0.250	**0.014**	1.0
EBC	0.783	0.125	N.D.
		**TLSP**	
Serum	0.359	0.496	0.6875
Nasal secretions	0.578	0.437	0.687
EBC	0.875	0.750	N.D.
		**TGF-β**	
Serum	0.898	0.275	1.0
Nasal secretions	0.218	0.269	0.125
EBC	N.D.	N.D.	N.D.
		**IL-3**	
Serum	0.721	0.059	0.148
Nasal secretions	0.250	0.396	0.437
EBC	N.D.	N.D.	N.D.
		**INF-γ**	
Serum	0.839	0.261	0.812
Nasal secretions	0.107	0.078	0.611
EBC	0.250	1.0	N.D.
		**IL-2**	

Serum	0.687	**0.031**	0.125
Nasal secretions	0.750	0.375	0.156
EBC	N.D.	N.D.	N.D.
**IL-33**			
Serum	1.0	0.500	1.0
Nasal secretions	1.0	0.498	1.0
EBC	N.D.	N.D.	N.D.

Abbreviations: EBC: Exhaled Breath Condensate; GM-CSF: Granulocyte-Macrophage Colony-Stimulating Factor; IFN-γ: Interferon gamma; IL: Interleukin; TGF-β: Transforming Growth Factor beta; TSLP: Thymic Stromal Lymphopoietin; N.D.: Not detectable or not determined. For IL-5, values reflect total IL-5 measured by the assay, which detects antibody-bound and free cytokine. In addition, the assay does not separate free from total IL-5 in all matrices.

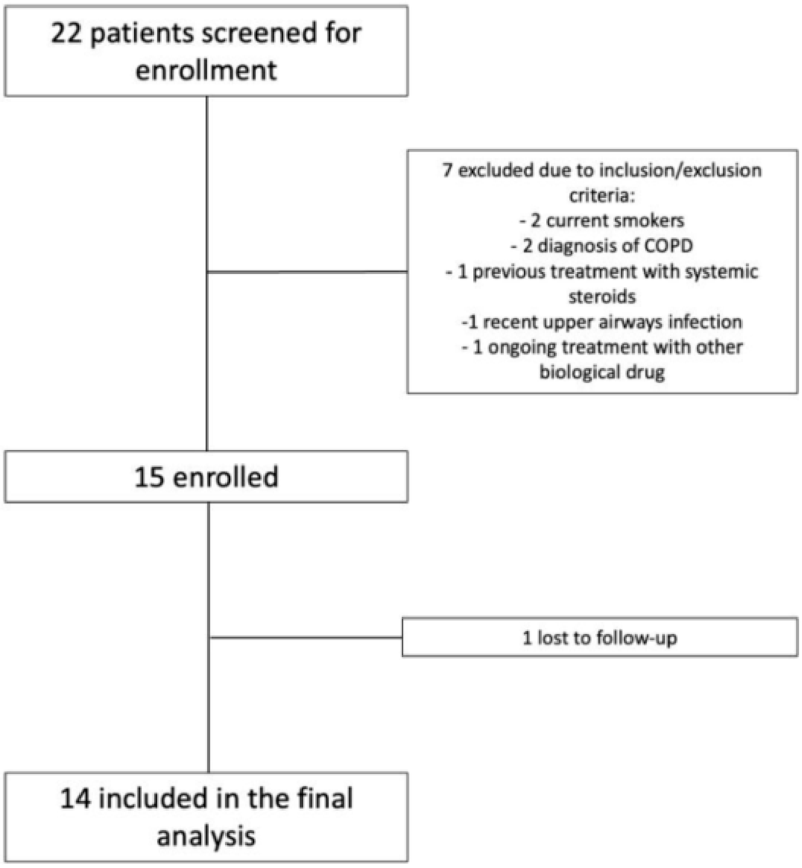

Supplemental Figure S1. Schematic flowchart of the enrollment process.

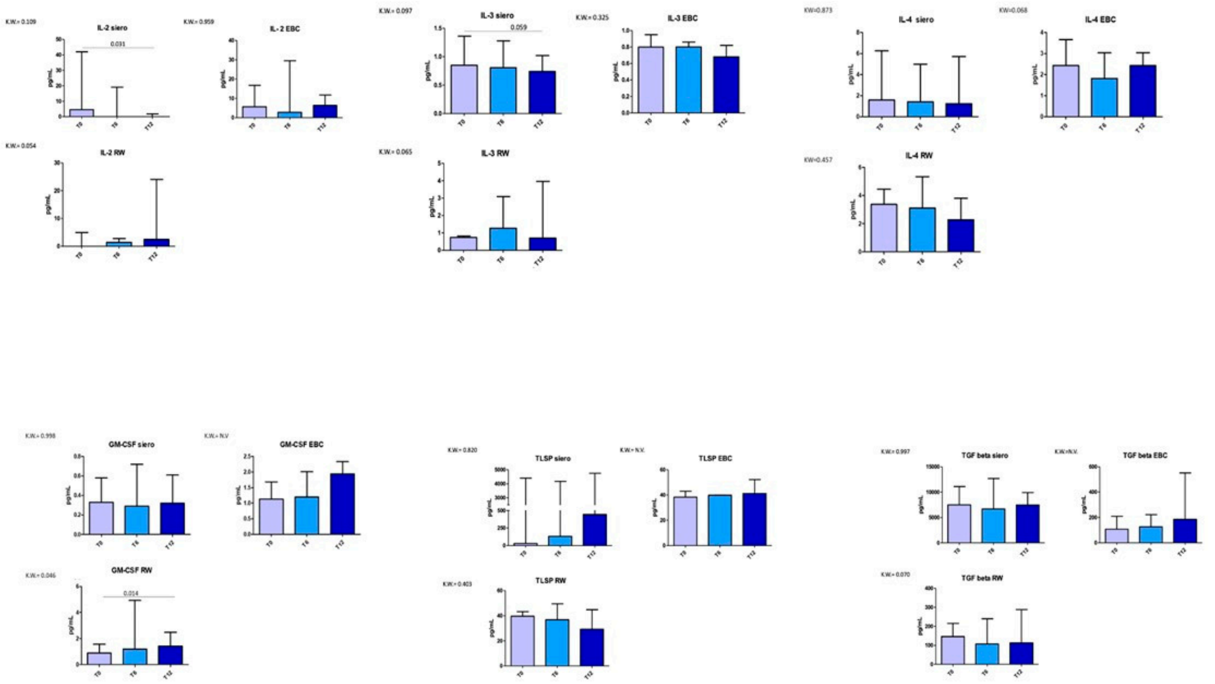

Supplemental Figure S2. Variations in Interleukin (IL)-2, IL-3, IL-4, Granulocyte-Macrophage Colony-Stimulating Factor (GM-CSF), Thymic Stromal Lymphopoietin (TSLP), and Transforming Growth Factor-beta (TGF-β) across timepoints (T_0, T_6, T_{12}) in different biological matrices, including serum, exhaled breath condensate (EBC), nasal secretions, and concentrated nasal secretions. KW: Kruskal–Wallis P-values.
